# Pediatric Usher Syndrome Type 2A with Coexisting Rheumatic Heart Disease and Upper Gastro-Intestinal Bleed: A Case Report

**DOI:** 10.31729/jnma.8842

**Published:** 2024-12-31

**Authors:** Bishal Yadav, Tunam Khadka, Toyendra Jung Shah, Mandish Prasad Phuyal, Rajesh Lamichane, Bikash Chaurasiya

**Affiliations:** 1 Department of Pediatrics, Kanti Children's Hospital, Maharajgunj, Kathmandu, Nepal; 2 Kathmandu Medical College, Sinamangal, Kathmanu, Nepal; 3 Department of Medicine, Grande International Hospital, Dhapasi, Kathmandu, Nepal

**Keywords:** *case report*, *pediatric*, *retinitis pigmentosa*, *usher syndrome*

## Abstract

Usher syndrome is a rare autosomal recessive disorder characterized by progressive sensorineural hearing loss and retinitis pigmentosa, typically present from birth and later symptoms, including loss of night vision and peripheral vision slowly progressing to blindness. The condition exhibits clinical and genetic diversity and currently lacks the definitive treatment. This report presents a case of a ten-year-old female diagnosed with Usher syndrome type IIA via whole exome sequencing. The delayed onset of visual symptoms often leads to a misdiagnosis to isolated deafness in early years. The early identification allows for better prognosis through surveillance and intervention in hearing and visual impairments. If usher patients can receive a timely diagnosis, genetic molecular therapies may help preserve the photoreceptors, subsequently development of blindness could be delayed or possibly be prevented.

## INTRODUCTION

Usher syndrome is a genetic disorder with autosomal recessive pattern of inheritance characterized by progressive sensorineural hearing loss and retinitis pigmentosa (RP). It is a major cause of deaf-blindness, with the prevalence of 2.2 per 100,000 population.^1,2^ Clinical features are varied significantly, making it necessary for careful diagnosis and genetic confirmation. Early identification of the condition allows better prognosis, through surveillance and intervention for hearing and visual impairments.^3^ The delayed onset of visual symptoms often leads to misdiagnosis of isolated deafness in early years.^4^ We present a case of a ten-year-old female with Usher syndrome type IIA (USH2A) diagnosed by whole exome sequencing (WES).

## CASE REPORT

A ten-year-old female presented to the emergency unit with fever (104°F), dizziness, left upper abdominal pain, and multiple episodes of blood-stained vomiting for one day. She is a known case of Rheumatic Heart Disease, diagnosed two months ago, and has been on prednisolone and Penicillin V since diagnosis.

Additionally, she reported decreased hearing in the right ear since four months, suggestive of a possible associated auditory complication. On arrival, she was hypotensive, with a blood pressure reading indicative of shock, but her systemic examination findings were unremarkable, with no other overt abnormalities detected.

**Figure 1:**
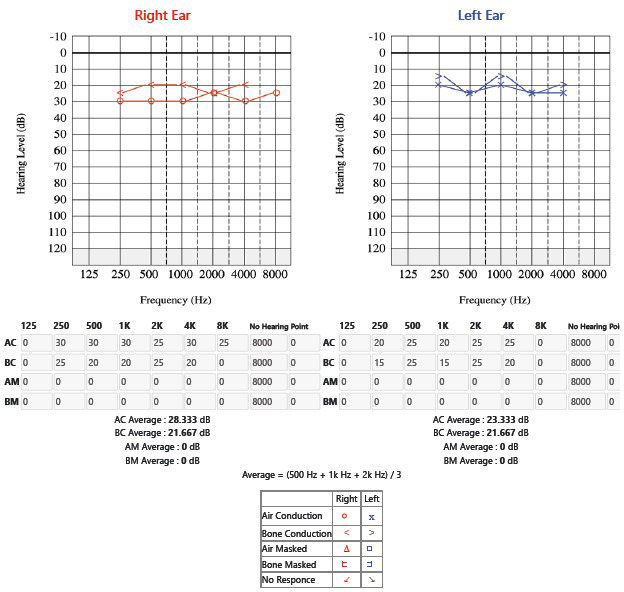
Pure tone audiometry showing right sided mild sensorineural hearing loss.

Laboratory findings revealed an ASO titer of 320 IU/mL, high sensitivity C-reactive protein (hsCRP) of 2.9 mg/dL, elevated ESR of 50 mm per hour, and positive stool occult blood test, while renal and liver function tests were within normal limits. ECG indicated prolonged QT interval. The patient was admitted to the Pediatric ICU for upper gastrointestinal bleeding and was treated with intravenous Pantoprazole and antibiotics. Steroids were discontinued due to suspected steroid toxicity. Audiological examination identified mild sensorineural hearing impairment (28.333 dB HL in the right ear and 23.333 dB HL in the left ear) (Figure 1) and bilateral As-type tympanogram was found. Echocardiography revealed severe mitral regurgitation, mild aortic regurgitation, and moderate pulmonary artery hypertension with a left ventricular ejection fraction of 60%. Gastrointestinal evaluation, including endoscopy, showed normal findings. Given the history of RHD and SNHL, we suspected a genetic condition or associated syndrome and proceeded with genetic testing. Genetic testing was done via whole exome sequencing confirmed the diagnosis of Usher syndrome type 2A.

Ophthalmological consultation revealed no signs of retinitis pigmentosa or steroid toxicity during the examination, providing reassurance regarding potential ocular complications. A follow-up evaluation was scheduled for six months to monitor any future developments. The patient was discharged with a prescription for Penicillin V and Enalapril to manage her underlying conditions. Regular follow-ups were advised in cardiology, ophthalmology & ENT every three months in outpatient departments to ensure comprehensive monitoring and management of her health.

## DISCUSSION

Usher Syndrome is classified into four main subtypes USH types I, II, III and IV; depending on the severity of hearing impairment, vestibular dysfunction, and the age at onset of retinitis pigmentosa (RP), which are further subclassified into 11 subtypes associated with an autosomal recessive mutation in a specific gene.^1^ The gene encoding Usher syndrome type IIA (USH2A) is located on chromosome 1q41 and spans 800 kb. It has 72 exons and two alternatively spliced transcripts.^5^

The USH2A gene is responsible for producing two known isoforms of the usherin protein.^4^ This protein plays a crucial role in the development and proper functioning of cochlear hair cells in the inner ear, which are essential for hearing. Additionally, usherin is vital for the long-term maintenance and survival of retinal photoreceptors, the specialized cells in the retina that detect light and enable vision. Mutations in the USH2A gene can disrupt these processes, leading to hearing loss and progressive vision impairment.^4^ Usher syndrome, particularly type 2A, presents with moderate congenital sensorineural hearing impairment and a gradual onset of retinitis pigmentosa, distinguishing it from that of type 1, which features early-onset RP and severe hearing impairment.^6^ Hearing loss is generally identified in childhood; however, RP occurs most often during the second decade of life, leading to delay in the diagnosis of USH.^7^ Since our patient was only ten years old, there was absence of RP. Patients with USH2A have an autosomal recessive inheritance pattern, however pseudo-dominant inheritance has also been observed.^5^

Usher syndrome type II should be suspected in individuals with; congenital sensorineural hearing loss that is mild to moderate in the low frequencies and severe to profound in the higher frequencies, intact or variable vestibular responses, RP and a family history consistent with autosomal recessive inheritance. The "sloping" audiogram is a hallmark feature of USH2. While the hearing loss itself is typically stable, individuals may perceive it as worsening over time due to a decline in speech perception, possibly linked to reduced vision, which hampers subconscious lip-reading abilities. For most individuals with USH2, hearing aids provide sufficient support. However, cochlear implants are highly effective for those whose speech and sentence recognition tests show limited improvement with hearing aids.^8^ If clinical characteristics are unclear, the diagnosis is established by identifying biallelic pathogenic mutations in one of three genes: ADGRV1 (adhesion G protein-coupled receptor V1), USH2A (usherin), or WHRN (whirlin).^8^ Whole exome sequencing (WES) analysis can be done to hunt for the disease-causing mutations.^9^

In many affected people, replacing or repairing damaged gene copies may be feasible with a correct genetic diagnosis for Usher syndrome while novel treatments are being refined.^10^ Patients with USH primarily rely on early diagnosis and counseling to adjust to dual sensory loss. If usher patients can receive a timely diagnosis, genetic molecular therapies may help preserve the photoreceptors, subsequently development of blindness could be delayed or possibly be prevented. Even when total prevention cannot be achieved, delay in disease progression can still improve the quality of life for patients.^4^ Interdisciplinary management, involving cardiology, ENT & ophthalmology, is crucial in improving patient outcomes and addressing the multi-system challenges presented by Usher syndrome.

In conclusion, Usher Syndrome is a genetically diverse autosomal recessive disorder that leads to combined hearing and vision loss, profoundly impacting patients' lives. Research has revealed that USH proteins are crucial for hair cell development and mechanotransduction, with some mutations causing nonsyndromic hearing loss by selectively affecting these processes. It is important to understand the pathologic mechanisms of USH for advancing diagnosis, prevention, and treatment strategies.
